# Supplementary material for: An improved organ explant culture method reveals stem cell lineage dynamics in the adult Drosophila intestine
Source: eLife. 2022 Aug 25;11:e76010. doi: 10.7554/eLife.76010 (PMC9578704; doi:10.7554/eLife.76010)
Supplement: Transparent reporting form [file elife-76010-transrepform1.pdf]

Westbrook Centre
Milton Road
Cambridge CB4 1YG, UK
P 01223 855340
W elifesciences.org
T @elife

eLife's transparent reporting form

We encourage authors to provide detailed information *within their submission* to facilitate the interpretation and replication of experiments. Authors can upload supporting documentation to indicate the use of appropriate reporting guidelines for health-related research (see EQUATOR Network), life science research (see the BioSharing Information Resource), or the ARRIVE guidelines for reporting work involving animal research. Where applicable, authors should refer to any relevant reporting standards documents in this form.

If you have any questions, please consult our Journal Policies and/or contact us: editorial@elifesciences.org.

Sample-size estimation

- You should state whether an appropriate sample size was computed when the study was being designed
- You should state the statistical method of sample size computation and any required assumptions
- If no explicit power analysis was used, you should describe how you decided what sample (replicate) size (number) to use

Please outline where this information can be found within the submission (e.g., sections or figure legends), or explain why this information doesn't apply to your submission:

> As our work focused on the development of a novel live-imaging technique, sample size was not statistically estimated prior to experiments. For each biological phenomenon we analysed, all observable events were quantified and included in our statistical analyses. Sample sizes were therefore the result of the need to obtain enough data points for proper statistical analysis.

Replicates

- You should report how often each experiment was performed
- You should include a definition of biological versus technical replication
- The data obtained should be provided and sufficient information should be provided to indicate the number of independent biological and/or technical replicates
- If you encountered any outliers, you should describe how these were handled
- Criteria for exclusion/inclusion of data should be clearly stated
- High-throughput sequence data should be uploaded before submission, with a private link for reviewers provided (these are available from both GEO and ArrayExpress)

Please outline where this information can be found within the submission (e.g., sections or figure legends), or explain why this information doesn't apply to your submission:

> For each experimental condition, data was collected from ≥4 separate intestines imaged in ≥2 different live-imaging sessions. Unique biological events of interest from all imaged intestines were defined as biological replicates. Repeated measurements of the same unique event were defined as technical replicates, instead. All data referenced in figures are described in the provided source data files. Criteria for inclusion/exclusion of data points and outliers handling are described in the Results and Methods sections of the submission.

eLife Sciences Publications, Ltd is a limited liability non-profit non-stock corporation incorporated in the State of Delaware, USA, with company number 5030732, and is registered in the UK with company number FC030576 and branch number BR015634 at the address Westbrook Centre, Milton Road, Cambridge CB4 1YG.

Statistical reporting

- Statistical analysis methods should be described and justified
- Raw data should be presented in figures whenever informative to do so (typically when N per group is less than 10)
- For each experiment, you should identify the statistical tests used, exact values of N, definitions of center, methods of multiple test correction, and dispersion and precision measures (e.g., mean, median, SD, SEM, confidence intervals; and, for the major substantive results, a measure of effect size (e.g., Pearson's r, Cohen's d)
- Report exact p-values wherever possible alongside the summary statistics and 95% confidence intervals. These should be reported for all key questions and not only when the p-value is less than 0.05.

Please outline where this information can be found within the submission (e.g., sections or figure legends), or explain why this information doesn't apply to your submission:

> All statistical analyses shown are described in the relevant figure legends, including statistical methods used, number of replicates, and corrections for multiple testing.

(For large datasets, or papers with a very large number of statistical tests, you may upload a single table file with tests, Ns, etc., with reference to sections in the manuscript.)

Group allocation

- Indicate how samples were allocated into experimental groups (in the case of clinical studies, please specify allocation to treatment method); if randomization was used, please also state if restricted randomization was applied
- Indicate if masking was used during group allocation, data collection and/or data analysis

Please outline where this information can be found within the submission (e.g., sections or figure legends), or explain why this information doesn't apply to your submission:

> Experimental groups were defined based on treatment (e.g. control-fed vs SDS-fed flies) or genetic background of the live-imaging specimens analysed, or based on biological distinctions (*e.g.* symmetric vs asymmetric division events). All relevant sample groupings are described in the main text and figure legends.

Additional data files ("source data")

- We encourage you to upload relevant additional data files, such as numerical data that are represented as a graph in a figure, or as a summary table
- Where provided, these should be in the most useful format, and they can be uploaded as "Source data" files linked to a main figure or table
- Include model definition files including the full list of parameters used
- Include code used for data analysis (e.g., R, MatLab)
- Avoid stating that data files are "available upon request"

Please indicate the figures or tables for which source data files have been provided:

eLife Sciences Publications, Ltd is a limited liability non-profit non-stock corporation incorporated in the State of Delaware, USA, with company number 5030732, and is registered in the UK with company number FC030576 and branch number BR015634 at the address Westbrook Centre, Milton Road, Cambridge CB4 1YG.

All source data for analyses described in figures are attached as source data files referenced for the figure of origin.

A supplementary file ("Supplementary File 1.xlsx") is also included to simplify calculations needed for the formulation of the custom culture medium we describe in the main text.

Lastly, three source code files (two Python scripts and an ImageJ macro) are included in this submission. These annotated scripts were used in our analysis pipeline for local image registration ("Source code 1.py"), cell/lineage tracking ("Source code 2.ijm"), and lineage reconstruction and data summarization ("Source code 3.py").

eLife Sciences Publications, Ltd is a limited liability non-profit non-stock corporation incorporated in the State of Delaware, USA, with company number 5030732, and is registered in the UK with company number FC030576 and branch number BR015634 at the address Westbrook Centre, Milton Road, Cambridge CB4 1YG.
